# General practitioners must acquire skills to communicate with child with Autism Spectrum Disorder to regain their values and role in the follow-up – phenomenological study

**DOI:** 10.1080/02813432.2021.1913905

**Published:** 2021-06-21

**Authors:** Bernard Clary, Eva Marengo-Sorli, Agnès Oude-Engberink, Elodie Million, Sylvain Pavageau, Michel Amouyal, Philippe Serayet, François Carbonnel, Gérard Bourrel, Béatrice Lognos

**Affiliations:** a Department of General Practice, Montpellier University, Montpellier, France; b Institut Desbrest d’Epidémiologie et de Santé Publique (IDESP), Montpellier University, Montpellier, France; c Laënnec General Practice Center, Trèbes, France; d Avicenne Multiprofessional Health Centre, Cabestany, France

**Keywords:** Autism Spectrum Disorder (ASD), mental disorders, family practice, general practitioner, qualitative research, children

## Abstract

**Objective:**

To understand the perceptions and attitudes of general practitioners (GPs) regarding children with an Autism Spectrum Disorder (ASD).

**Design:**

Phenomenological qualitative study.

**Setting:**

Three focus groups, clinical settings.

**Subjects:**

French GPs.

**Main outcome measures:**

22 GPs took part in the study divided among three focus groups. They were volunteers to participate. Data were transcribed verbatim and analysed using a grounded theory data analysis, completed with a semiopragmatic analysis.

**Results:**

Representing autism as a strange disorder in the doctor–patient relationship, GPs perceive a loss of sensory contact with the child with ASD that prevents the usual professional relationship between doctor and patient. They disengage themselves from monitoring the subject, concentrating on supporting the family. According to them, their role was to refer the patient to a specialist in the case of clinical intuition, but they have several reasons to give themselves time, all the more so because once the diagnosis is made, they lose sight of the patient and their place in the care pathway. GPs expressed the need to acquire skills and strategies to communicate with the autistic child to recover their role and values.

**Conclusion:**

GPs are disconcerted by the idea of communicating with children with ASD, as it takes them out of their usual professional benchmarks. They need communication tools that enable them to regain their role and relational value of the patient-centred approach. Beyond this, the question of the ‘ethics of care’ of the patient with a joint attention disorder is raised.

**KEY POINTS**
- GPs are disconcerted with the idea of communicating with children with ASD.
- GPs need communication tools that enable them to regain their role and relational value of the patient-centred approach.
- The question of the ‘ethics of care’ of the patient with a joint attention disorder is raised.

## Background

### The general framework

Autism is a general behavioural disorder of early onset, before the age of 3 years, and has an estimated prevalence of 62 in 10,000 children under the age of 18 [1]. The concept of autism has evolved considerably since its first description [2,3]. The Diagnostic and Statistical Manual of Mental Disorders-V (DSM-V) defines Autism Spectrum Disorders (ASDs) and includes them with neurodevelopmental disorders [4].

In the case of clinical suspicion in primary care, different tests can be carried out depending on age and the presence or absence of a developmental intelligence disorder [5–7]. Guidelines vary from one country to another [7–11].

In France, children are subject to 20 mandatory medical examinations between birth and 6 years of age by paediatricians or general practitioners (GPs). The objectives include the early detection of neurodevelopmental disorders. French guidelines insist on the need for multidisciplinary collaboration using validated tools to make the diagnosis [12]. Unfortunately, the considerable increase in demand for child psychiatric care has lengthened the waiting period for a consultation with a specialist team [6]. Parents thus find themselves alone with their child for a long time and turn to their GP, confronted with the question of diagnosis and daily management.

In 2014, Venkat et al. noted that for physicians encountering patients with ASD, the combination of altered social interactions, communication difficulties and stereotyped behaviours creates an additional barrier to the diagnosis and treatment of these individuals. They added that careful preparation of the examination environment, direct involvement of caregivers and the patient, and the use of communication techniques and pharmacologic adjuncts could help physicians treat the patient with an ASD on an outpatient basis [13].

In 2020, Couglhan et al. showed that GPs had mixed knowledge and experience in identifying autism and managing the care of children with autism. At one end of the continuum were GPs who had not heard of autism or who endorsed outdated etiological theories. On the other hand, GPs had a good knowledge of the disorder but limited confidence in their ability to identify it [14]. Thus, the GMs are concerned. In the medical literature, studies on the care of children with ASD by GRs focus more on their knowledge than on their perceptions, attitudes, or expectations [15,16]. In fact, we did not find any studies devoted exclusively to the last three points.

In the medical literature, studies on the care of children with ASD by GPs focus more on their knowledge than on their perceptions, attitudes, or expectations [15,16]. Therefore, we did not find any studies devoted exclusively to the last three points.

Understanding how GP’s stand with these children, how much they invest in this relationship and manage it could provide the keys for improving care.

The objective of this study was to understand the different perceptions and attitudes of GPs toward children with ASDs based on their lived experience.

## Methods

### Type of study

We used a semiopragmatic phenomenological approach in accordance with our objective of exploring the lived experience of GP’s.

### Recruitment

The data collection was carried out by collecting the verbatims of three focus groups that were set up. The GPs participating in the focus groups were recruited from continuing medical education peer groups in the Occitania Region. These three focus group meetings were held on 13 May, 19 June, and 20 October 2013. A total of 22 volunteer GPs, five women and 17 men, aged 28–62 years, participated.

The focus groups met for 1h45 (9 doctors), 1h17 (8 doctors) and 1h09 (5 doctors), respectively. The groups were composed of physicians belonging to the same continuing medical education peer group, which facilitated authentic expression.

### Data collection

The focus group chose to collect the data to be able to benefit from wider information using peers’ group dynamics.

The phenomenological guide is a person-centred guide exploring four dimensions: (1) representations of GPs, (2) lived experience of a situation (memorisation of a case and how it was experienced), (3) their role and (4)/needs (Table 1).

**Table 1.** Interview guide on the subject of the care of patients presenting with an autism spectrum disorder.

1. When the term autism is mentioned, what first comes to mind? 2. Do you remember a situation in which you were confronted with an autistic child? 2.1. What was your experience of this situation? Do you remember what you thought, felt or did? Do you experience the same thing with other chronic conditions, or in your opinion is there a difference? In your opinion, what makes it a particular experience? Do these patients have specific health problems? 2.2. Have you ever experienced a situation in which you doubted the existence of autism? With which specific symptoms (or autism spectrum disorder) did you have this doubt? What did you feel about this idea? Could you tell me about it? 2.3. Do you feel comfortable talking to the parents about this, or is it difficult for you? 2.4. Have you experienced a situation in which the parents spoke to you about doubts they had regarding their child? What happened? 3. What role do you think you played in these situations in which the diagnosis of autism is made, and what role should you have? 4. In your opinion, is it easy contacted the healthcare professionals who work with these children? 5. How do you explain that the subject of autism has been more common in recent years than before? 6. In a condition such as autism, what do you think you need to better manage the condition, or do you feel that this management doesn’t really concern you directly beyond any intercurrent pathologies?

The focus groups had been structured by two researchers (EMS and BC) (Table 2). The facilitator (BC) facilitated the groups while respecting participants' freedom of expression without directing the meaning of what they said. The recordings were made by MCS, using a smartphone for the audio and a digital camera, then the verbatim transcriptions were made. The film was screened to consider the elements of non-verbal communication.

**Table 2.** Characteristics of the participants.

Age				
	25–35 years	35–45 years	45–55 years	>55 years
Number	4	4	2	12
Sector of activity				
	Rural	Semi-rural	Urban	Rural
Number	0	12	10	0

Before questioning the participants, the host collected consent from the participants to record the proceedings. All volunteers were present. All participants were able to express themselves. There was no payment.

We followed the criteria of the SRQR [17] and the COREQ [18] to write this paper.

### Data analysis

We conducted a semiopragmatic study. It is a specific phenomenological approach based on C.S. Peirce’s theories [19] and is a descriptive method for categorising lived experiences recorded in interview transcripts. In this method, first the analyst considers all the semiotic elements of a text, including linguistic and contextual clues. Second, after selecting themes that are relevant to the research question, the analyst assembles them to create emerging first-level categories. Third, empirical general categories emerge by a continuous comparison process. Simultaneously, semiopragmatic data interpretation procedure inspired by C.S. Peirce’s class of signs theory allows these emerging categories to be logically ordered [20]. Typically, as a result of this ordering, the conceptually densest category (i.e. of the highest level in the hierarchy of signs) commands the meaning of the phenomenon at play [21]. Table 3 summarises the steps of the method used [22].

**Table 3.** Steps of a pragmatic phenomenological analysis [22].

Word by word transcription of recordings (verbatim).
A reading using a floating attention, followed by a focussed reading.
Extracting signifying units from the text and grouping these units by themes.
Collating textual and contextual meaningful semiotic elements and their semio-pragmatic characterisation.
A first categorisation through a regrouping of these semiotic elements and of the signifying units in accordance with the research question.
Enriching the categories by continuing comparison until theoretical saturation is reached.
Placing the emerging categories in logical order and reducing them and their properties in order to model the ensemble in integrative semio-pragmatic statements.

## Results

### Participant characteristics

Each of the three focus groups lasted between 69 and 105 min. Participants described their personal lived experience freely. Their ages ranged from 28 to 62 years. Other characteristics are summarised in (Table 3).

### Semio-pragmatic phenomenological analysis

This is a continuous comparison procedure that allows the emerging categories to be enriched to the highest density. Four phenomenological statements emerged from the analysis.

#### Representations

*For the GPs, autism was a strange disorder of doctor–patient relationship*

As the GPs were unable to satisfy themselves with a complex and poorly operational definition of autism, they proposed definitions linked to their personal experience using vague terms: ‘a sort of shapeless entity, where nothing is certain’ said M10; for M20, ‘it’s a knot of incomprehension’; ‘a bubble’ for M7. According to GPs the mechanisms of autism were complex with many classifications: ‘there are dozens of different classifications of autism’ (M5).

#### Lived experience

*GPs perceive a loss of sensory contact with the child with ASD that prevents the usual professional relationship between doctor and patient. They compensate by investing their professional attitude to support the family*

GPs cannot perform their examinations with usual professional benchmarks as a patient-centred approach, which confuses them and makes their work more difficult. A GP cannot listen to a child who does not speak and does not make eye contact and cannot touch the ASD child**.** Several doctors noted ‘difficulty in making contact, making visual contact’. This led to feelings of discomfort, fear, refusal, and impotence, and also played a part in the fact that they did not name the ASD using its official definition. In these conditions, there is no clinical examination of the child: (M18) ‘Did you try and make contact with the child?’ Children with communication disorders disappear from the reference framework of GPs. Autistic children were ‘outside’ the professional doctor–patient relationship. M17: ‘Something is missing for me to really be the attending physician’.

During consultations, the GPs even tried to restore a ‘normal’ medical setting that would be reassuring for themselves and the parents, and which could lead them to a pseudo-examination that reassured the mother: ‘when I manage to put my hand on her tummy, without her getting frightened, well, her parents are happy’ (M7).

In extreme cases, the consultation was composed only of the relationship with the parents, with no clinical examination of the child: (M18) ‘contact’ and ‘I listened to the mother, because she was there’ answered M22.

The doctors, while they were unable to treat the children as they would have liked, were able to take on their role of ‘doctor’ (M8, M1, M14), support and companion for the parents. M18 defined his role as that ‘of reassuring and comforting people in their role, even though they were doing a great job’. They thus felt compassion and admiration for the parents and saw themselves ‘playing a role in the entourage… Yes… Because we see the problems, the concerns of people in relation to the child: and my grandson… and my nephew… What can we do for him?’ (M13).

At the same time, they recognised the expert role of the family: ‘they’re the ones who’re right, not me…’ (M11).

#### Their role

*According to them, their role is to refer the patient to a specialist in case of clinical intuition, but they have several reasons to give themselves time, all the more so because once the diagnosis is made, they lose sight of the patient and their place in the care pathway.*

The GPs considered that their place in the care system was not clearly defined. This role consisted of mentioning an intuitive diagnosis when faced with a communication disorder, for early orientation towards specialised structures: ‘you have an intuition and can make the referral easily, if there is a doubt’ (M5). The doctors confirmed that they took longer than the 3 months recommended for acting or announcing the diagnosis:

- out of fear of the consequences of the diagnosis on the parents,
- to avoid stigmatisation
- because, in their opinion, it did not seem to modify the child’s prognosis in any way.

On the contrary, they were more comfortable with eliminating the diagnosis.

Less GPs criticise the lack of coordination between the different specialists (neurologists, paediatricians) once the diagnosis has been made. They did not find a place for themselves in the midst of all the different actors: ‘Once the diagnosis has been made, we’re really in the background’, said M8.

#### Needs

*GPs expressed the need to acquire skills and strategies to communicate with the autistic child to recover their role and values.*

GPs did not ask for clarification of the diagnostic classifications of autism but expressed a need to know how to manage their involvement as shown by the following response: M5 ‘I don’t need to know that there are dozens of different classifications of autism, that’s no use to me at all’. Once the child has been oriented, the doctor played only a marginal role in the care pathway and was only asked to intervene in cases of intercurrent pathologies from general medicine or administrative problems.

However, the GPs struggled in these cases of common practice, like M6 who ‘dreaded clinical examinations…’ always wondering, ‘how I was going to tame him’. Several doctors expressed ‘the need to understand the particular communication modes’ (M4) and to know how to approach these children in cases of intercurrent pathologies, stressing the difficulty in examining ‘a child who isn’t present’ (M19).

## Discussion

### Statement of principal findings

The semio-pragmatic phenomenological qualitative approach allowed us to achieve our goal of understanding how GPs experience the relationship with an autistic child, their perceptions, and attitudes during follow-up. Representing autism as a strange disorder of doctor–patient relationship, GPs perceive a loss of sensory contact with the child with ASD that prevents the usual professional relationship between doctor and patient. Faced with the loss of sensory contact with the autistic child, GPs lose their professional value based on the doctor–patient relationship and disengage themselves from monitoring the subject, concentrating on supporting the family. According to them, their role is to refer the patient to a specialist in case of clinical intuition, but they have several reasons to give themselves time, all the more so because once the diagnosis is made, they lose sight of the patient and their place in the care pathway. GPs expressed the need to acquire skills and strategies to communicate with the autistic child to recover their role and values.

### Strengths and weaknesses of the study

Despite a selection bias (some participants might not have participated if it were not for members of their peer group), our choice of peer group focus groups allowed physicians to explain their experience in an authentic way.

Of the participants, women were under-represented: 22% of the GPs were women versus 33% on average in 2013 according to the French Direction for research, studies, evaluations, and statistics.

The internal validity was consolidated by triangulation of the researchers: after each focus group, different analysts (EMS, BC) compared their results. There was no external validation by the participants. The theoretical saturation of the categories was achieved, with the third focus group providing only the properties of the emerging categories. In our sample, if we had included the doctors who had received training and GPs who did not participate in a peer group, so as to have a sample with more solid maximum variation, the validity of our results would have been improved and possible new strategies might have emerged.

### Findings in relation to other studies

#### About their representations

The representation of these children by the GPs may have been biased by the lack of knowledge of the disease found also in the literature review by Tatlow-Golden et al. [16].

#### About the lived experience

GPs practice is based on interpersonal exchanges and aims for a patient-centred approach [23]. Lykke K et al. insist on the fact that GPs based their assessments of child development and well-being on the physical examination, the child’s behaviour during the consultation, and communication with the child more than on the conversation with the parents [24]. Faced with the loss of sensory contact with the autistic child, GPs lose their professional value based on patient-centred approach and disengage themselves from monitoring the subject, concentrating on supporting the family. In fact, according to O Solomon et al., although there are three primary social actors (in the medical consultation with a child with ASD), the interaction between them cannot be categorised as triadic, as is the case in the TD children’s visits. Rather, there are three separate dyadic interactions – child–mother, child–doctor, and mother–doctor – that take place during most of the visits described here [25]. GPs in the study highlighted two main semiological obstacles: the communication disorder and the ‘joint attention disorder’ [26]. In the absence of ‘joint attention’, that is, without being able to share the child’s attention (a triadic relationship), the doctor ‘disengaged’ from an ineffective relationship in the face of a patient ‘who was not there’ [27]. This result highlights the principle stated by Shea, which makes the ‘engagement process’ between the doctor and patient the cement of a good doctor–patient relationship and ultimately of a therapeutic alliance [28]. These concepts can be linked to Gernsbacher’s ‘lack of reciprocity’. This author suggests that the clinicians have forgotten that reciprocity (with children with ASDs) must be mutual and symmetrical [29]. This finding is consistent with NICE's recommendations and Lee, which calls for psychosocial interventions to improve joint attention [30,31].

#### About their roles

Another result is the delegation of expertise to the family of which GPs become the support, which corresponds to the results of Gaillard et al. who evoked the development of ‘expert knowledge’ by the parents [32]. As a result, GPs did not feel they had any legitimacy in posing a ‘diagnostic label’ or in being ‘references’ for the follow-up of their chronic disease. GPs do not use scales in their practices; the Modified Checklist for Autism in Toddlers (M-CHAT), was known by only one doctor out of 15 in the study by de Fenekile [33]. They do not know of any tools to improve communication with the ASD’s people. Finally, they did not know of a clearly defined care pathway for the follow-up of the autistic person [14]. If we put our results into the perspective of French guidelines, we can see that the GPs refer their patients easily when there is a behavioural disorder. They justified the delay in announcing the diagnosis for three reasons: uncertainty regarding the diagnosis at the beginning, the fear of stigmatisation with the impact on the parents, and the idea that, once the diagnosis has been made, it makes no difference to the prognosis, contrary to scientific data [12,34]. Before the age of 18 months, new French clinical practice guidelines [12] offer the term of diagnosis of neurodevelopmental disorder, which is more appropriate due to the difficulties of a formal diagnosis and the heterogeneous and non-specific nature of early developmental trajectories.

The disappearance of ASD from the field of vision of GPs meant that they were only contacted occasionally for intercurrent pathologies prevalent in many of these children [35].

#### About their needs

Finally, and perhaps this is what emerges clearly from our study, GPs emphasise the imperative need to be able to do their job, being responsible for the ‘health of the child’. To do this, they say they need tools or strategies adapted to regain good communication with the autistic child. Samel and Luterman have proposed a method ‘See-Hear-Feel-Speak’, an approach conducive to learning with the goal of enabling clinicians and their teams to facilitate patient-centred encounters with paediatric patients with ASD [36].

The child disappeared a second time, objectively this time, swept up in the specialist care network from which GPs are excluded. GPs felt they had been dispossessed of their function of continuity and coordination of care. They did not receive any information from the network caring for the child. Shared information and care would improve their skills for dealing with autistic patients as recommended by the Scottish and French guidelines [10,12].

### Meaning of the study: an ethical perspective

GPs must not withdraw from the follow-up of autistic patients when they are called upon to do so. The study shows that they are concerned about being able to take care of the autistic child as well as others, but that they are looking for tools to help them. According to Levinas [37], ‘the face [of the Other] speaks to me and thereby invites me to a relation incommensurate with a power exercised, be it enjoyment or knowledge’. How can a doctor exercise the ‘ethics of recognition’, when ‘the Other is absent?’ This could concern other illnesses with joint attention disorders, such as dementia (Alzheimer’s disease, Tay–Sachs disease, and locked-in syndrome). Accepting that the inner life of the Other exists, regardless of the way in which it presents, is an essential preliminary to care [38].

## Conclusion

This qualitative study provides a better understanding of the difficulty of appropriating recommendations for ASDs in children and adolescents. It highlights that GPs are confused by the idea of communicating with children with ASDs, as it takes them out of their usual professional bearings. It raises the issue of their disengagement from the diagnosis and coordinated follow-up of these children in primary care. GPs need communication tools that enable them to regain their role and relational value of the patient-centred approach. The question of the ‘ethics of recognition’ in pathologies with joint attention deficit disorder is raised. Clinical trials must be conducted in the context of primary care.
